# *Changes in International Lawmaking: Actors, Processes, Impact. *Conference Report of the 16th Annual Meeting of the European Society of International Law (ESIL), held in Stockholm from 9 to 11 September 2021.

**DOI:** 10.1007/s12399-022-00891-y

**Published:** 2022-04-12

**Authors:** Hubert Mayer

**Affiliations:** Fakultät für Wirtschafts- und Organisationswissenschaften, Professur für öffentliches Recht, Universität der Bundeswehr München, Werner-Heisenberg-Weg 36, 85577 Neubiberg, Germany

## Opening of a hybrid conference

Changes in international lawmaking brought about by new actors and processes were the theme of the Annual ESIL Meeting, held in Stockholm. For the first time, due to the pandemic, the conference was organized in a hybrid format, with more online participants than on-site attendants; itself already a powerful sign of change. Crown Princess Victoria of Sweden opened the conference. She recalled that Stockholm was, in 1972, the site of the 1st United Nations World Conference on the Environment (UNCHE), which adopted the Stockholm Declaration (United Nations 1973); the United Nations, she said, have always been a cornerstone of Swedish foreign policy, with Dag Hammarskjöld and Folke Bernadotte representing it. The Crown Princess, herself a peace and conflict researcher, demonstrated her personal interest in the subject by attending further panels on the first day of the conference.

Jessika van der Sluis, Dean of the host law faculty at Stockholm University, emphasized the paramount importance of the rule of law in international relations, especially for small and medium-sized industrialized nations like Sweden. The role of states in the production of international law is changing, said Photini Pazartzis, President of ESIL, private and transnational actors are emerging, and the conference programme reflects this increasing diversity. According to Hans Corell, former Under-Secretary-General for Legal Affairs and the Legal Counsel of the United Nations (1994–2004), the greatest enemy of the rule of law is corruption; the former is indispensable for justice, stability and security. The rule of law is now threatened even in democracies such as Poland. The Raoul Wallenberg Institute of Human Rights and Humanitarian Law (RWI) provides politicians worldwide with an informative guide to the rule of law in 26 languages (RWI 2012). Pål Wrange (Stockholm) spoke on behalf of the organizers. Although the Covid-19 pandemic affects everyone, not everyone is affected equally. The challenges for governments and international law are immense, especially in terms of sovereignty, universality and solidarity. In terms of the sheer volume of regulations and numerous new actors, international lawmaking under the classical Westphalian system had been comparatively easy, unlike today; in the meantime, even rules that were not recognized by the international community of states as binding hard law would very much have an impact on international law; however, all this did not mean the end of the Westphalian system, it had been under pressure at all times and yet always survived.

## The politics of global lawmaking

Sarah Nouwen (EUI Florence) and Marrti Koskenniemi (Helsinki) discussed the current political implications of global lawmaking against the background of historical developments. Property and sovereignty are the “yin and yang” of international law and its generous gift to the respective holders of positions of power, but this no longer applies exclusively to states; in this respect, one should no longer postulate the ideal type of the state-centered Westphalian system. According to Nouwen, decision-making and regulation processes in international law now also take place very much on the corporate side, beyond the sphere of sovereignty, a process whose channels of influence and decision-making need to be made (more) transparent. Furthermore, the mere invocation of the binding force of formally correct norms is no longer sufficient in a world marked by global injustice; international law and politics are inextricably interwoven; one must ask much more consciously about the winners and losers of international regulations and take a more conscious look at the diversity of the different perspectives on (international) law, for which deeper encounters are indispensable to promote mutual understanding.

Koskenniemi asked about the historical premises for the development of international law in Europe (Koskenniemi 2021). He stated that a turn towards legal concepts such as property (and later on sovereignty) had taken place during the 13th century by recourse to ancient Roman law, which was attractive for aspiring rulers, because it could help to overcome prevailing theological constraints. In a world of sinners, ruling over others, expropriation, and the establishment of new property (including through appropriation of the fruits of [others’] labor) could thus be justified and, not least, used to counter popular demands of the Franciscan reform movement, which propagated equal common property and solidarity with the poor. Instead, the adopted ancient Roman contract and commerce law guaranteed merchants security and stability for their burgeoning trades. Even non-lawyers such as Francisco de Vitoria and Immanuel Kant, despite their disdain for the profession, would have deliberately resorted to legal concepts in order to be able to position themselves against theological doctrines. Once the principle of sovereignty had been established, law had fallen behind economics around the 18th and 19th century, for sovereignty alone said nothing about how to use the power it entailed; the doctrines of economics had taken over this task as a guiding principle for the modernizing law.

## On the deformalization of international law

For Concepción Escobar Hernández (International Law Commission), guiding principles, action plans, codes of conducts, etc., as legal texts informally adopted free of mandatory procedural and formal requirements, are an undeniable expression of the increasing complexity of international law; they are also undoubtedly norms in the sociological and political sense, but ultimately non-binding under international law for lack of a sufficient state consensus. Without a minimum of procedural formality, one could not speak of binding law. Nevertheless, this category of norms was not without influence on international law; thus, the establishment of new binding law could be triggered or existing law could be further developed by way of interpretation. Ultimately, however, the reasons for this type of informal law-creation were predominantly of a non-normative nature, namely considerations of flexibility and efficiency. According to Anna Leander (IHEID Geneva), the trend towards increasingly deformalized law and rule-making is closely linked to the changes in government forms: however, we also need to think more carefully about what is actually meant by ‘deformalization’; in her view, the focus has so far been too actor-centered, and there is an underexposed material side to this phenomenon. As examples, she cited the role of documents in international financial law or the embedding of code-based technologies in everyday life. Law is undergoing massive change at its margins, she said, noting a sociological transformation through the mediatization of law into a technological element. States are not opposing this process, but involved in it, yet the necessary public discussion does not take place, the commercially practice-relevant formation of norms also takes place precisely by avoiding a general debate. The phenomenon of this deformalization of law requires further, more in-depth conceptualization and discussion.

## International and subnational lawmaking from below

Globally positioned and networked activists deliberately use national jurisdictions as an arena to initiate and drive global change, as the example of the LGBTQIA* movement vividly demonstrates. According to Balakrishnan Rajagopal (MIT), in order to better explore this dynamic and tense change due to the increasing influence of non-state actors on international lawmaking, the reference to the pluralization of institutional forums is more appropriate than the reference to legal pluralism, which is empirically undeniably given, but too state-centered and therefore analytically insufficient. Theories of international law also remain stuck in Westphalian categories, this even applies to feminist approaches or the Third World Approach to International Law (TWAIL). Pluralization encompasses political antagonisms between states and social movements, forms of institutional and extra-institutional disputes, hierarchical and pluralistic relationships, as well as the relationship between states of the Global North and the Global South. The latter is a key question, as one can observe the beginnings of a hegemonic power formation in the Global South. Regarding the issue of overcoming the climate crisis, the North increasingly forms the counter-hegemonic position.

Raffaela Kunz (MPI Heidelberg) spoke about the imminent danger of the research-based knowledge system being captured by powerful market players in science publishing. In order to narrow the knowledge gap, the innovative Open Science movement had aimed to transform the scientific publishing system with its oligopoly-like structures, high publication costs and exorbitant profit margins, driven by actors from the public sector such as universities, libraries and researchers, to whom a high degree of legitimacy is attributed. However, the results achieved by Project DEAL or Plan S, for example, tend to perpetuate the status quo and the power relations in the science publishing system, causing the knowledge gap to widen; moreover, there is a shift in charging, away from reading towards publishing, which leads to new exclusions. In South America, which pursues a different Open Access (OA) method, the worldwide dominance of the heavily criticised gold OA approach is feared and perceived as a neo-colonial instrument. The fundamental problem, she argues, is the lack of public and political accountability in the field of this informal norm-setting, compounded by the fact that the interests of dominant actors are even more powerful here than in the field of traditional international lawmaking.

Giedre Jokubauskaite (Glasgow) argued that not every participation of non-state actors in the norm-setting process is tantamount to lawmaking from below. The decisive factor, she said, was who could set the agenda and determine the debates; the dominant discourses were still those on security and the economy, with questions about the assumption of historical liability on the margins. The starting point was therefore the inclusion of representatives who were in a disadvantaged position, in order to be able to actually speak of legislation from below. The United Nations Declaration on the Rights of Peasants and Other People Working in Rural Areas (UNDROP) serves as an example; human rights were indispensable as a ‘living instrument’ in this area (UN General Assembly 2019). In another panel, Natalie Jones (Cambridge) referred to the participation of representatives of indigenous people in international negotiations; the decisive factor was that these representatives could refer to an (internal) democratic selection, an accreditation by the home state was not necessary, a justified participation was rather to be decided by the secretariats of the hosting international organizations. In addition to financial hurdles, there were also sectoral restrictions, for example, the participation of indigenous groups was generally limited to environmental and social issues, but much less to trade and investment.

Laura Prat (King’s College London), however, drew attention to new developments, particularly in the area of investment, with a view to South America. There, local referenda by affected groups on the legitimacy of investment projects by multinational corporations in the mining sector have become increasingly legally binding since 1999. Even smaller communities with no more than 6000 members could thus bring such large-scale investments by transnational corporations to an end. The balance of power and hierarchies has leveled out where the corporations no longer had any influence on determining local consent. It is a sub- as well as a multinational regulatory process with the participation of transnational actors, enriched by conflicting goals between (overall) economic development and (environmental) interests of the affected local population. (Mega)cities and metropolitan regions are key actors in the decentralized fight against climate change, Maša Kovič Dine (Ljubljana) said. The Edmonton Declaration signed by more than 3400 cities in North America, the Lima-Paris Action Agenda (LPAA) and the Paris Declaration supported by more than 100 cities worldwide are proof of this. Although the commitments vary, what they have in common is a commitment to more stringent commitments than those made by states, and a system based on city cooperation. This urban practice is a central element that can no longer be isolated from international lawmaking, and cities must therefore be much more involved in it as stakeholders.

## Remembering James Crawford (1948–2021)

Friends and companions commemorated James Crawford (ICJ), who died on 31 May 2021, with a special panel. Kaj Hobér (Uppsala) called Crawford a giant of international law, of formidable competence, and yet always pursuing a practical and pragmatic approach. He had always particularly appreciated his work as a judge in international commercial disputes because of the dynamics prevailing in this area of law. Laurence Boisson de Chazournes (Geneva) said that there were very few international lawyers whose first name alone was enough to know who they were talking about; his contributions to international law were indispensable readings (Crawford 2007, 2014, 2019), the rule of law was his concern, and he had also worked pro bono for this cause.

A visibly moved Alain Pellet (Paris Nanterre) paid tribute to his friend James Crawford – a modest man and firm Australian – as a fantastic team player and attentive listener, who was very interested in promoting young talent. Despite his training in common law, he had always been aware that international law was an interplay of continental law and common law. His sense of compromise had worked wonders in the adoption of the Draft Articles on the Responsibility of States for Internationally Wrongful Acts by the International Law Commission (ILC) in August 2001 (UN General Assembly 2002).

Peter Tomka (ICJ) also praised Crawford’s never-to-be-forgotten role as ILC’s last of five Special Rapporteurs in this regard, not least due to his pragmatic streamlining of the draft, including the abandonment of the controversial concept of ‘international crime’. Crawford’s view had been that the draft first had to prove itself in practice, which is why he had not pushed for the conclusion of a binding convention; he had been right in doing so, the draft articles on state responsibility had asserted itself, it was accepted by the states and meanwhile counted among the most cited international law documents in court. Unfortunately, he had not been able to work on the history of international law, which had been planned in three volumes.

In addition to Crawford’s immense dedication to his work, all speakers emphasized his collegiality, fairness, friendliness, his famous smile and his sense of family. It was unmistakable that the human loss was felt to be at least as great as the professional one.

## Is Covid-19 a gamechanger in international law?

For Bryan Mercurio (Hong Kong), the Covid-19 crisis does not represent a paradigm shift, it merely reinforces the already tangible departure from liberal principles in (economic) international law: firstly, a shift of production capacities back to the domestic economy is taking place, especially by large states, combined with increasing protectionism; in this respect, the pandemic is a welcome occasion to reduce dependencies on China within the global supply chain. Second, the willingness of states to engage in international lawmaking is declining, not least due to a lack of confidence, exacerbated by the uncertainties still associated with Covid-19. Thirdly, there is an increasing shift away from multilateral agreements towards bilateral treaties, which are also limited to specific sectors. In view of the major challenges posed by the Covid-19 crisis, this should have led to greater cooperation between states within the framework of multilateral agreements such as the World Trade Organization (WTO), but the opposite is the case. Instead, there will be more bilateral agreements in niche areas; in this respect, smaller states in particular will have to act and react skilfully. The Covid effect will have a significant impact on future international lawmaking. However, the WTO rules on intellectual property (TRIPS, Trade-Related Aspects of Intellectual Property Rights) would not hinder the necessary innovations; in the fight against the pandemic, it was the insufficient production of vaccines and not a lack of innovation that was the big problem in the first place; moreover, the pharmaceutical companies would have no interest in refusing licences for vaccine production. If Covid-19 has shown anything, it is the paramount importance of cooperation in the scientific field, not only in the development of vaccines, said Gian Luca Burci (IHEID Geneva). A science-policy interface must therefore be integrated into a future WHO agreement on pandemic prevention; the potential danger of a virus pandemic originating from mass animal husbandry, for example, affects cross-cutting problems of food production, animal husbandry and health, public health and, last but not least, economic interests. He did not expect a fundamental change in the rules on intellectual property, but waivers under the TRIPS Agreement could be considered.

Diane Desierto (Notre Dame) sees the danger of a normalization and proliferation of legal emergency regimes due to the pandemic. 109 states worldwide have enacted regulations on states of emergency. At the same time, democratic control mechanisms for reviewing these emergency measures would be weakened; should the Covid-19 pandemic become endemic, it would not be possible to remain in emergency mode permanently. Human rights activists would have to become more active and concern themselves more with (world) trade, economics and health, because at the moment too many restrictions are justified with reference to the pandemic. The pandemic also exposes inequalities and asymmetries: countries with the least access to vaccines are among the most affected by Covid-19. For Martin Scheinin (Oxford), the Covid-19 pandemic cannot be overcome without respecting human rights. The decisive factor is not how the virus behaves, but how people behave, which is why more emphasis must be placed on persuasion rather than on governmental instruction. Instead, overreactions and underreactions were observed in pandemic management. Unlike after 11 September 2001, there had been no power grab at the United Nations Security Council, although measures could be justified under Chapter VII of the UN Charter. Should a global agreement on pandemic prevention and control be reached, human rights would have to be included.

## Global law as the end of international law?

For Andrea Leiter (Amsterdam), a transnationally understood global law makes it possible to overcome the conceptual weaknesses of the classical doctrine of international law with its focus on states as the authoritative legal entities and creators of law (Art. 38 ICJ Statute). New concepts are urgently needed in this regard in order to be able to better meet the current challenges and shifts in power – not least through innovations in technology and digitalization – as well as the associated injustices; such a reconceptualization also by no means implies forgetting history. Anne Orford (Melbourne) was very sceptical about the characterization of international law as conservative in contrast to the allegedly dynamic nature of global law. One must ask themself who is propagating global law as a new category of thought and action, why and with which possibilities of influence; it also has to be examined who ultimately benefits from it. She rather sees the danger of re-colonialization if the consensus of states necessary for lawmaking in international law should lose importance in favor of private and transnational actors, especially since there is no international political institution that is constituted in a way that enables it to adequately represent and integrate all actors of global law. It is more important to fight for a different, a better international law.

For Makane Mbengue (Geneva), international law has never been purely ‘westphalian’, it has always been ‘global’, not least because of its evolutionary and adaptive nature. International law and global law would be mutually supportive, the latter being a means to an end in order to improve international law. As evidence, he pointed to the 2017 draft of a Global Pact for the Environmemt: stemming from a private initiative by authoritative lawyers, its modus operandi was clearly attributable to the sphere of influence of global law, but its goal was the deliberate strengthening, and not the replacement, of already existing (environmental) international law – now affirmed by a United Nations General Assembly resolution (UN General Assembly 2018; Le club des juristes 2017). Dire Tladi (Pretoria) also questioned the need for the new category of global law, noting that a greater role for non-state actors is already evident throughout international law, despite the insistence of structurally conservative states on respect for their national interests. So, even if we all look at the same international law, we always discover different aspects.

For him, the turn to global law can be explained by the unequal influence of the Global South on norm-setting in international law: at best, the new discourse reflects this old power imbalance; at worst, it obscures it once again.

The conference was excellently organized, with a benchmark high technological, human and financial effort. According to Pål Wrange, the hybrid conference format is here to stay, as is the coronavirus. If this should be the case, apart from the undeniably positive effects on the ecological footprint of international conferences, there are also questions of justice with regard to who will be able to participate on-site in such conferences in the future, when, how often, why and with what kind of financial support; notably, it could get more difficult for young academics to establish and maintain their own transnational networks (and friendships).

## Supplementary Information

German full-text translation of the Conference Report of the 16th Annual Meeting of the European Society of International Law (ESIL) by Hubert Mayer
