# Supplementary material for: Changes in International Lawmaking: Actors, Processes, Impact. Conference Report of the 16th Annual Meeting of the European Society of International Law (ESIL), held in Stockholm from 9 to 11 September 2021
Source: Z Außen Sicherheitspolit. 2022 Apr 12;15(1):97–104. doi: 10.1007/s12399-022-00891-y (PMC9002223; doi:10.1007/s12399-022-00891-y)
Supplement: Supplementary file 1 — German full-text translation of the Conference Report of the 16th Annual Meeting of the European Society of International Law (ESIL) by Hubert Mayer [file 12399_2022_891_MOESM1_ESM.pdf]

ZFAS
Zeitschrift für Außen- und Sicherheitspolitik

Changes in International Lawmaking: Actors, Processes, Impact. Tagungsbericht zur 16. Jahreskonferenz der European Society of International Law (ESIL)

Hubert Mayer

Angenommen: 03. Februar 2022

1 Eröffnung der hybriden Konferenz

Die durch neue Akteure[1] und Arbeitsweisen bewirkten Änderungsprozesse in der internationalen Normrechtsetzung waren das Thema der 16. Jahrestagung der European Society of International Law (ESIL), die vom 9.-11. September in Stockholm stattfand. Die Konferenz wurde pandemiebedingt erstmals hybrid organisiert und war mit mehr Teilnehmern online vor den heimischen Bildschirmen als Anwesenden vor Ort, ein nachdrückliches Zeichen von Wandel. Kronprinzessin Victoria von Schweden eröffnete die Veranstaltung offiziell. Sie verwies auf Stockholm als den Ort der ersten Weltumweltkonferenz der Vereinten Nationen (UNCHE), die 1972 mit der Verabschiedung der Stockholm Declaration endete (United Nations 1973); die Vereinten Nationen seien ein Eckstein schwedischer Außenpolitik, Dag Hammarskjöld und Folke Bernadotte stünden stellvertretend dafür. Ihr persönliches Interesse an der Materie bekundete die studierte Friedens- und Konfliktforscherin durch den Besuch weiterer Panels am ersten Konferenztag.

Jessika van der Sluis, Dekanin der gastgebenden juristischen Fakultät der Universität Stockholm, betonte die überragende Bedeutung der Herrschaft des Rechts in den internationalen Beziehungen gerade für kleinere und mittlere Industrienationen wie Schweden. Die Rolle der Staaten bei der Erzeugung von Völkerrecht ändere sich, so Photini Pazartzis,

[1] Im vorliegenden Artikel wird, abweichend vom ZfAS-Standard, bei personenbezogenen Substantiven die männliche grammatikalische Form verwendet. Der Autor schließt damit Personen jeden Geschlechts gleichermaßen ein.

Hubert Mayer
Fakultät für Wirtschafts- und Organisationswissenschaften, Universität der Bundeswehr München,
Werner-Heisenberg-Weg 36, 85577, Neubiberg, Deutschland
E-Mail: hubert.mayer@unibw.de

Präsidentin der ESIL, private und transnationale Akteure träten neu hinzu, das Konferenzprogramm spiegele diese zunehmende Diversität wider. Für Hans Corell, Untergeneralsekretär und leitender Rechtsberater der Vereinten Nationen von 1994 bis 2004, stellt Korruption den größten Feind der Rechtsstaatlichkeit dar; letztere sei unverzichtbar für Gerechtigkeit, Stabilität und Sicherheit. Die Herrschaft des Rechts sei mittlerweile selbst in Demokratien wie etwa Polen gefährdet. Das Raoul Wallenberg Institut (RWI) versehe Politiker weltweit in 26 Sprachen mit einem informativen Handlungsleitfaden zum Rechtsstaatsprinzip (RWI 2012). Pål Wrange (Stockholm) sprach für die Veranstalter. Auch wenn das Covid-19 Virus alle betreffe, so seien doch nicht alle gleich davon betroffen. Die Herausforderungen für die Regierungen und das Völkerrecht seien immens, gerade in Bezug auf Souveränität, Universalität und Solidarität. Angesichts der schieren Menge an Regelungen und zahlreicher neuer Akteure sei die internationale Rechtsetzung im früher dominant westfälisch geprägten internationalen System anders als heute vergleichsweise leicht gewesen; mittlerweile würden auch Regeln, die von der Staatengemeinschaft nicht als verbindliche *Hard Laws* anerkannt seien, sehr wohl auf das Völkerrecht zurückwirken; all dies bedeute aber nicht das Ende des westfälischen Modells, es habe zu allen Zeiten unter Druck gestanden und doch stets überlebt.

2 Die Politik globaler Normrechtsetzung

Sarah Nouwen (EUI Florenz) und Marrti Koskenniemi (Helsinki) diskutierten über die aktuellen politischen Implikationen globaler Normsetzung vor dem Hintergrund historischer Entwicklungen. Eigentum und Souveränität seien das Yin und Yang des Völkerrechts und dessen großzügige Morgengabe an die jeweiligen Inhaber von Machtpositionen, was aber nicht mehr ausschließlich nur auf Staaten zutreffe; man dürfe insofern nicht länger vom Idealtypus des staatenzentrierten westfälischen Systems ausgehen. Völkerrechtspolitische Entscheidungs- und Regulierungsprozesse fänden jenseits der Souveränitätssphäre mittlerweile sehr stark auch auf Unternehmensseite statt, ein Prozess, dessen Einfluss- und Entscheidungskanäle transparent(er) gemacht werden müssten, so Nouwen; auch die bloße Berufung auf die Verbindlichkeit formell korrekt gesetzter Normen genüge in einer von globalen Ungerechtigkeiten geprägten Welt nicht mehr, Völkerrecht und Politik seien untrennbar miteinander verwoben, man müsse viel sorgfältiger nach den Gewinnern und Verlierern internationaler Regelungen fragen und bewusster die Diversität der unterschiedlichen Perspektiven auf das (Völker-)Recht in den Blick nehmen, wozu zur gegenseitigen

Verständnisförderung vertieftere Begegnungen unabdingbar seien. Koskenniemi fragte nach den historischen Prämissen für das sich entwickelnde Völkerrecht und konstatierte für das 13. Jahrhundert (durch den Rückgriff auf das antike römische Recht) eine Wendung hin zu Rechtsbegriffen (wie Eigentum und Souveränität), attraktiv für aufstrebende Machthaber, weil sich somit Zwänge vorherrschender theologischer Konzepte überwinden ließen; in einer Welt von Sündern konnte so die Herrschaft über andere, die Enteignung und die Begründung neuen Eigentums (auch durch die Aneignung der Früchte [fremder] Arbeit) gerechtfertigt und nicht zuletzt den populären Forderungen der franziskanischen Reformbewegung entgegensetzt werden, die ein gleichberechtigtes Gemeineigentum und die Solidarität mit den Armen propagierte (Koskenniemi 2021). Das rezipierte römische Vertrags- und Handelsrecht garantierte stattdessen Kaufleuten Sicherheit und Stabilität für ihr aufstrebendes Gewerbe. Auch gerade Nicht-Juristen wie Francisco de Vitoria und Immanuel Kant hätten trotz ihrer Geringschätzung für den Berufsstand bewusst auf Rechtsbegriffe zurückgegriffen, um sie gegen theologische Doktrinen in Stellung bringen zu können. Nachdem das Prinzip der Souveränität einmal etabliert war, sei das Recht gegenüber der Ökonomie um das 18./19. Jahrhundert herum ins Hintertreffen geraten, denn Souveränität allein sage noch nichts darüber aus, wie die damit verbundene Macht zu nutzen sei, die Lehren der Ökonomie hätten diese Aufgabe als Leitbild für das sich modernisierende Recht übernommen.

3 Zur Entformalisierung des Völkerrechts

Für Concepción Escobar Hernández (International Law Commission) sind Guiding Principles, Action Plans, Code of Conducts und ähnliches als frei von zwingenden Verfahrens- und Formvorschriften informell beschlossene Rechtstexte unbestreitbarer Ausdruck der zunehmenden Komplexität des Völkerrechts; sie seien auch zweifellos Normen im soziologischen und politischen Sinn, doch letztlich völkerrechtlich unverbindlich mangels eines hinreichenden Staatenkonsenses. Ohne ein Mindestmaß an prozeduraler Förmlichkeit könne man nicht von verbindlichem Recht sprechen. Gleichwohl sei diese Normkategorie nicht ohne Einfluss auf das Völkerrecht; so könnte dadurch die Begründung neuen, verbindlichen Rechts angestoßen bzw. geltendes Recht im Wege der Auslegung weiterentwickelt werden. Letztlich seien aber die Gründe für diese Art informeller Rechtschöpfung überwiegend nicht-normativer Art, nämlich Flexibilitäts- und Effizienzerwägungen. Die Hinwendung zu einer zunehmend entformalisierten Rechts- und Regelsetzung sei eng verbunden mit den sich ändernden Handlungsformen der Staaten und Regierungen, so Anna Leander (IHEID Genf): Man müsse

aber auch genauer darüber nachdenken, was eigentlich unter Deformalisierung zu verstehen sei; ihr sei der Fokus bislang zu sehr akteurszentriert, es gäbe eine unterbelichtete materielle Seite dieses Phänomens. Als Beispiele nannte sie die Rolle von Urkunden im internationalen Finanzrecht oder die Einbettung codebasierter Technologien in den Alltag. Das Recht ändere sich gerade massiv an seinen Rändern, sie konstatiere hier einen soziologischen Wandel durch die Mediatisierung des Rechts in ein technisches Element. Die Staaten stünden diesem Prozess nicht etwa entgegen, sondern seien darin eingebunden, die notwendige öffentliche Diskussion finde dennoch nicht statt, die kommerziell praxisrelevante Normformierung erfolge auch gerade durch das Vermeiden einer Debatte. Das Phänomen dieser Entformalisierung des Rechts bedürfe weiterer, vertiefter Konzeptualisierung und Diskussion.

4 Internationale und subnationale Rechtsetzung von unten

Global aufgestellte und vernetzte Aktivisten nutzten gezielt nationale Gerichtsbarkeiten als Arena, um einen weltweiten Wandel anstoßen und vorantreiben zu können, wie das Beispiel der LGBTQIA*-Bewegung anschaulich zeige. Um diesen dynamischen und spannungsgeladenen Wandel durch den zunehmenden Einfluss nicht staatlicher Akteure auf die internationale Rechtsetzung besser erkunden zu können, sei die Bezugnahme auf die Pluralisierung institutioneller Foren eher geeignet als der Verweis auf den Rechtspluralismus, der empirisch unbestreitbar gegeben, aber zu sehr staatenzentriert und deshalb analytisch unzureichend sei, so Balakrishnan Rajagopal vom Massachusetts Institute of Technology (MIT). Theorien des internationalen Rechts blieben ebenfalls westfälischen Kategorien verhaftet, dies gelte selbst für feministische Ansätze oder den Third World Approach to International Law (TWAIL). Diese Pluralisierung umfasse politische Gegensätze zwischen Staaten und sozialen Bewegungen, Formen institutioneller und außerinstitutioneller Auseinandersetzungen, hierarchische und pluralistische Beziehungen sowie das Verhältnis zwischen Staaten des globalen Nordens und des globalen Südens; letzteres sei eine Schlüsselfrage, man könne Ansätze einer Hegemonialmachtbildung im Globalen Süden beobachten, denn bei der Frage um Bewältigung der Klimakrise bilde der Norden zunehmend die gegenhegemoniale Position.

Raffaela Kunz (Max-Planck-Institut Heidelberg) sprach über die drohende Gefahr der Kaperung des forschungsbasierten Wissenssystems durch marktmächtige Akteure im Wissenschaftsverlagswesen. Die Open Science Bewegung habe zur Verkleinerung der Wissenskluft die Transformation des wissenschaftlichen Publikationssystems mit seinen

oligopolähnlichen Strukturen, hohen Publikationskosten und exorbitanten Gewinnmargen, zum Ziel gehabt, vorangetrieben durch Akteure aus dem öffentlichen Bereich wie Universitäten, Bibliotheken und Forscher, denen ein hohes Maß an Legitimität zugeschrieben werde. Doch die im Rahmen z. B. des Project DEAL oder Plans S erzielten Resultate führten eher zu einer Perpetuierung des Status quo und der Machtverhältnisse im wissenschaftlichen Veröffentlichungswesen, so dass die Wissenskluft eher noch vergrößert würde; zudem finde eine Verlagerung der Gebührenerhebung weg von der Lektüre hin zur Veröffentlichung statt, was zu neuen Exklusionen führe. In Südamerika, das eine andere Open-Access Methode verfolge, werde die weltweite Dominanz des stark kritisierten Gold-Open-Access Ansatz befürchtet und als neokoloniales Instrument wahrgenommen. Das grundsätzliche Problem sei der Mangel an einer öffentlichen und politischen Rechenschaftspflicht im Bereich dieser informellen Normsetzung, verstärkt um die Tatsache, dass die Interessen der marktbeherrschenden Akteure hier noch wirkmächtiger seien als im Bereich der herkömmlichen internationalen Rechtsetzung.

Nicht jede Beteiligung nicht staatlicher Akteure im Normierungsprozess komme einer Rechtsetzung von unten gleich, so Giedre Jokubauskaite (Glasgow). Entscheidend sei, wer die Agenda setzen und Debatten bestimmen könne; dominant seien nach wie vor westlich geprägte Diskurse über Sicherheit und Ökonomie, am Rande stünden Fragen zur Übernahme historischer Verantwortung. Ausgangspunkt sei deshalb die Einbeziehung von Vertretern, die sich in einer benachteiligten Position befänden, um tatsächlich von einer Rechtsetzung von unten sprechen zu können. Als Beispiel diene die United Nations Declaration on the Rights of Peasants and Other People Working in Rural Areas (UNDROP). Die Menschenrechte seien in diesem Bereich als „Living Instrument" unverzichtbar (United Nations 2019). Auf einem weiteren Panel verwies Natalie Jones (Cambridge) auf die Teilnahme von Vertretern indigener Völker an internationalen Verhandlungen; entscheidend sei, dass sich diese Repräsentanten auf eine (binnen-) demokratische Auswahl berufen könnten, eine Akkreditierung durch den Heimatstaat sei nicht erforderlich, über eine gerechtfertigte Beteiligung sei vielmehr durch die Sekretariate der internationalen Organisationen zu entscheiden. Neben finanziellen Hürden gäbe es aber auch sektorale Restriktionen, so beschränke sich die Beteiligung indigener Gruppen in der Regel auf Umwelt- und Sozialfragen, weit weniger aber auf Handel und Investitionen.

Auf neue Entwicklungen gerade im Investitionsbereich machte aber Laura Prat (King's College London) mit Blick auf Südamerika aufmerksam. Dort gewönnen seit 1999 lokale Referenden durch betroffene Gruppen über die Legitimität von Investitionsvorhaben multinationaler Konzerne im Minenbereich immer mehr an rechtsverbindlicher Bedeutung. Selbst kleinere Gemeinschaften mit nicht mehr als 6000 Mitgliedern könnten dadurch solche

Großinvestitionen transnationaler Konzerne zu Fall bringen. Die Machtverhältnisse und Hierarchien hätten sich dort eingeebnet, wo die Konzerne keinen Einfluss mehr auf die Ermittlung der Zustimmung vor Ort hätten. Es handle sich um einen sub- sowie gleichzeitig um einen multinationale Regelungsprozess unter Beteiligung transnationaler Akteure, angereichert durch Zielkonflikte zwischen (gesamt-)wirtschaftlicher Entwicklung und (Umweltschutz-)Interessen der betroffenen Bevölkerung vor Ort. (Mega)Städte und Metropolregionen seien maßgebliche Akteure bei der dezentralen Bekämpfung des Klimawandels, so Maša Kovič Dine (Ljubljana). Die von mehr als 3400 Städten in Nordamerika unterzeichnete Edmonton Declaration, die Lima-Paris Action Agenda (LPAA) sowie die von weltweit mehr als 100 Städten unterstützte Paris Declaration seien Beleg dafür. Auch wenn die Verpflichtungszusagen variieren würden, gemeinsam sei ihnen die Festlegung auf striktere Zusagen als die von den Staaten eingegangenen Verpflichtungen sowie ein auf Städte-Kooperationen angelegtes System. Diese Städtepraxis sei ein zentrales Element, das von der Völkerrechtsetzung nicht mehr isoliert werden könne. Die Städte müssten deshalb als Interessensvertreter darin viel stärker einbezogen werden.

5 Gedenken an James Crawford (1948-2021)

Freunde und Weggefährten gedachten dem am 31.Mai 2021 verstorbenen James Crawford (IGH) mit einem eigenen Panel. Kaj Hobér (Uppsala) nannte Crawford einen Giganten des Völkerrechts, von geradezu respekteinflößender Kompetenz, dabei stets einen praktischen und pragmatischen Ansatz verfolgend. Aufgrund der in diesem Rechtsbereich vorherrschenden Dynamik habe er seine Tätigkeit als Richter in Investitionsschutzstreitigkeiten immer besonders geschätzt. Es gäbe nur sehr wenige Völkerrechtler, bei denen allein die Nennung des Vornamens genüge, um zu wissen von wem man spreche, so Laurence Boisson de Chazournes (Genf); seine Beiträge zum Völkerrecht seien unverzichtbare Lektüre, die Herrschaft des Rechts war sein Anliegen, dafür habe er auch pro bono gearbeitet (Crawford 2006, 2014, 2019).

Ein sichtlich bewegter Alain Pellet (Paris Nanterre) würdigte seinen Freund James Crawford als fantastischen Teamplayer und aufmerksamen Zuhörer, dem viel an der Förderung junger Talente gelegen habe. Trotz seiner Ausbildung im Common Law sei ihm immer bewusst gewesen, dass das Völkerrecht ein Wechselspiel aus kontinentaleuropäischem Recht und anglo-amerikanischem Common Law sei. Sein Sinn für Kompromisse habe bei der Verabschiedung des Artikelentwurfs für die Verantwortlichkeit von Staaten für völkerrechtswidriges Handeln Wunder bewirkt (UN General Assembly 2002). Peter Tomka (IGH) lobte diesbezüglich

ebenfalls die Rolle Crawfords, nicht zuletzt durch dessen pragmatische Verschlankung des Entwurfs mitsamt der Abkehr vom umstrittenen Konzept des *International Crime*. Crawfords Ansicht sei es gewesen, dass sich der Entwurf zunächst in der Praxis bewähren müsse, deshalb habe er auch nicht auf den Abschluss einer verbindlichen Konvention gedrängt; er habe damit richtig gelegen, der Artikelentwurf zur Staatenverantwortlichkeit habe sich behauptet, er werde von den Staaten angenommen und zähle mittlerweile bei Gericht zu den am meisten zitierten Völkerrechtsdokumenten. Ein auf drei Bände angelegtes Werk zur Geschichte des Völkerrechts habe er leider nicht mehr in Angriff nehmen können. Alle Redner hoben neben Crawfords immens hohen Arbeitseinsatz seine Kollegialität, Fairness, Freundlichkeit sowie sein berühmtes Lächeln und seinen Familiensinn hervor. Unverkennbar war, dass der menschliche Verlust als mindestens ebenso groß empfunden wurde wie der fachliche.

6 Covid-19 als Zeitenwende im Völkerrecht?

Für Bryan Mercurio (Hong Kong) stellt die Covid-19-Krise keinen Paradigmenwechsel dar, sie verstärke lediglich die ohnehin bereits greifbare Abkehr von liberalen Prinzipien im (Wirtschafts-)Völkerrecht: Erstens erfolge eine Rückverlagerung von Produktionskapazitäten in die heimische Volkswirtschaft gerade durch große Staaten, verbunden mit einem zunehmenden Protektionismus; die Pandemie sei insofern willkommener Anlass, Abhängigkeiten von China innerhalb der globalen Lieferkette zu verringern. Zweitens sinke die Bereitschaft der Staaten, sich im Rahmen der internationalen Rechtsetzung zu engagieren, nicht zuletzt aufgrund fehlenden Vertrauens, noch einmal verstärkt durch die mit Covid-19 immer noch einhergehenden Ungewissheiten. Drittens finde eine zunehmende Abkehr von multilateralen Abkommen hin zu bilateralen Verträgen statt, die sich zudem auf spezielle Sektoren beschränkten. Eigentlich hätte angesichts der großen Herausforderungen durch die Krise dies zu einer größeren Kooperation der Staaten im Rahmen multilateraler Übereinkommen wie der WTO führen müssen, das Gegenteil sei der Fall. Stattdessen werde es zu mehr bilateralen Vereinbarungen in Nischenbereichen kommen; gerade kleinere Staaten müssten insofern geschickt agieren und reagieren.

Der *Covid-Effekt* werde ganz erhebliche Auswirkungen auf die künftige Rechtsetzung im Völkerrecht haben. Die WTO-Regeln zum geistigen Eigentum (TRIPS) würden die notwendigen Innovationen jedoch nicht behindern, bei der Pandemiebekämpfung sei zunächst die unzureichende Produktion von Impfstoffen und nicht etwa fehlende Innovation das große

Problem gewesen; die Pharmafirmen hätten zudem kein Interesse, Lizenzen zur Impfstoffproduktion zu verweigern. Wenn Covid-19 etwas aufgezeigt habe, dann die überragende Bedeutung von Kooperationen im Wissenschaftsbereich, nicht nur bei der Entwicklung von Impfstoffen, so Gian Luca Burci (IHEID Genf). In ein künftiges WHO-Abkommen zur Pandemieprävention müsse deshalb unbedingt eine Schnittstelle Wissenschaftspolitik integriert werden; die potentielle Gefahr einer Viruspandemie aus dem Bereich der Massentierhaltung betreffe z. B. Querschnittsprobleme der Lebensmittelproduktion, der Tierhaltung und -gesundheit, des öffentlichen Gesundheitswesens und nicht zuletzt wirtschaftliche Interessen. Von einer grundlegenden Änderung bei den Regeln über das geistige Eigentum gehe er nicht aus, es kämen aber Ausnahmegenehmigungen im Rahmen des TRIPS-Abkommens in Betracht.

Diane Desierto (Notre Dame) sieht die Gefahr einer Normalisierung und Proliferation rechtlicher Notstandsregime durch die Pandemie, weltweit hätten 109 Staaten Regelungen zum Ausnahmezustand in Kraft gesetzt. Gleichzeitig würden dadurch demokratische Kontrollmechanismen zur Überprüfung dieser Notfallmaßnahmen geschwächt; sollte die Covid-19 Pandemie endemisch werden, so könne man nicht dauerhaft im Notstandsmodus verweilen. Menschenrechtler müssten aktiver werden und sich mehr mit (Welt-)Handel, Wirtschaft und Gesundheit beschäftigen, denn momentan würden dort allzu viele Einschränkungen mit Verweis auf die Pandemie gerechtfertigt. Die Pandemie lege auch verstärkt Ungleichheiten und Asymmetrien offen: So seien die Staaten mit dem geringsten Zugang zu Impfstoffen am stärksten von Covid-19 betroffen. Für Martin Scheinin (Oxford) kann die Covid-19 Pandemie nicht ohne Einhaltung der Menschenrechte überwunden werden. Entscheidend sei nicht, wie sich das Virus verhalte, sondern die Menschen, deshalb müsse auch mehr Gewicht auf Überzeugungsarbeit statt auf staatliche Anweisungspolitik gelegt werden. Stattdessen habe man bei der Pandemiebewältigung Über- und Unterreaktionen beobachten können. Anders als nach dem 11. September 2001 sei es zu keiner Machtkonzentration beim Sicherheitsrat der Vereinten Nationen gekommen, obwohl Maßnahmen nach Kapitel VII der UN-Charta zu rechtfertigen wären. Sollte es zu einem globalen Abkommen zur Pandemieprävention und -bekämpfung kommen, müssten Menschenrechte zwingend einbezogen werden.

7 Globales Recht als Schlusspunkt des Völkerrechts?

Für Andrea Leiter (Amsterdam) ermöglicht ein transnational verstandenes Globales Recht die konzeptionellen Schwächen der klassischen Völkerrechtsdoktrin mit ihrem Fokus auf den Staaten als den maßgeblichen Rechtsträgern und Rechtsschöpfern (Art. 38 IGH-Statut) zu überwinden. Neue Konzepte seien diesbezüglich dringend notwendig, um den gegenwärtigen Herausforderungen und Machtverschiebungen – nicht zuletzt durch Innovationen bei Technik und Digitalisierung − sowie den damit einhergehenden Ungerechtigkeiten besser begegnen zu können; eine solche Rekonzeptionalisierung bedeute auch keineswegs Geschichtsvergessenheit. Anne Orford (Melbourne) war sehr skeptisch bezüglich der Charakterisierung des Völkerrechts als konservativ im Gegensatz zur vorgeblich dynamischen Natur des Globalen Rechts. Man müsse sich schon fragen, wer weshalb mit welchen Einflussmöglichkeiten das Globale Recht als neue Denk- und Handlungskategorie propagiere; auch sei zu prüfen, wer letztlich davon profitiere. Sie sehe eher die Gefahr einer Rekolonialisierung, wenn der zur Rechtsetzung im Völkerrecht notwendige Staatenkonsens zugunsten privater und transnationaler Akteure an Bedeutung verlieren sollte, zumal es international keine politische Institution gäbe, die so beschaffen sei, um alle Akteure des Globalen Rechts angemessen repräsentieren und einbinden zu können. Wichtiger sei es für ein anderes, ein besseres Völkerrecht zu kämpfen.

Für Makane Mbengue (Genf) war Völkerrecht niemals rein „westfälisch" geprägt, es sei schon immer auch „global" gewesen, nicht zuletzt aufgrund seiner evolutiven und auch anpassungsfähigen Natur. Völkerrecht und Globales Recht würden sich gegenseitig stützen, letzteres sei Mittel zum Zweck, um das Völkerrecht zu verbessern. Zum Beleg verwies er auf den Entwurf eines Global Pact for the Environment aus dem Jahr 2017: auf eine Privatinitiative maßgeblicher Juristen zurückgehend, sei dessen Modus Operandi eindeutig der Einflusssphäre des Globalen Rechts zuzuschreiben, doch sei dessen Ziel die bewusste Stärkung, und nicht die Ersetzung, des bereits existierenden (Umwelt)Völkerrechts – inzwischen bekräftigt durch eine Resolution der Generalversammlung der Vereinten Nationen (UN General Assembly 2018; Le club des juristes 2017). Dire Tladi (Pretoria) hinterfragte ebenfalls die Notwendigkeit der neuen Kategorie des Globalen Rechts; es lasse sich bereits jetzt im gesamten Völkerrecht eine größere Rolle für nichtstaatliche Akteure feststellen, und das trotz des Beharrens der strukturkonservativen Staaten auf der Beachtung ihrer nationalen Interessen. Selbst wenn wir alle dasselbe Völkerrecht anschauen würden, entdeckten wir doch immer unterschiedliche Aspekte. Die Hinwendung zum Globalen Recht erkläre sich für ihn aus dem ungleichen Einfluss des globalen Südens auf die Normsetzung im Völkerrecht: im besten Falle reflektiere der neue Diskurs dieses alte Machtgefälle, im schlechtesten Falle verschleiere er es erneut.

Die Tagung selbst war hervorragend organisiert, mit maßstabsetzend hohem technischen, personellen und finanziellen Aufwand. Das hybride Konferenzformat werde ebenso bleiben wie das Corona Virus, so Wrange. Wenn dem so sein sollte, dann stellen sich neben unbestreitbar positiven Auswirkungen hinsichtlich des ökologischen Fingerabdrucks internationaler Tagungen aber auch Gerechtigkeitsfragen dahingehend, wer zukünftig wann, wie oft, weshalb und mit welcher finanziellen Unterstützung direkt vor Ort an solchen Konferenzen teilnehmen kann; gerade für den wissenschaftlichen Nachwuchs könnte es schwieriger werden, eigene transnationale Netzwerke (und auch Freundschaften) begründen und pflegen zu können.

Literatur

Crawford, J. (2006). *The creation of states in international law.* Oxford: Oxford University Press.

Crawford. J. (2014). *Chance, order, change. The course of international law.* Leiden: Martinus Nijhoff Publisher.

Crawford, J. (2019). *Brownlie's principle of public international law.* Oxford: Oxfrod University Press.

Koskenniemi, M. (2021). *To the uttermost parts of the World: Ledal imagination and international power 1300 – 1870.* Cambridge: Cambridge University Press.

Le club des juristes (2017). Draft project. Global pact for the environment. https://www.iucn.org/sites/dev/files/content/documents/draft-project-of-the-global-pact-for-the-environment.pdf. Accessed 26 Oct 2021.

RWI – Raoul Wallenberg Institute (2012). Rule of law. A guide for politicans. https://rwi.lu.se/app/uploads/2012/09/Rule-of-Law-a-guide-for-politicians.pdf . Zugegriffen: 28. Okt. 2021.

UN – United Nations (1973). Report of the United Nations Conference on the human environment. https://undocs.org/en/A/CONF.48/14/Rev.1. Accessed 26 Oct 2021.

UN General Assembly (2002). Resolution adopted by the General Assembly on the responsibility of states for internationally wrongful acts.

UN General Assembly (2018). Resolution adopted by the General Assembly. Towards a global pact for the environment.

https://globalpactenvironment.org/uploads/Resolution10mai2018EN.pdf. Accessed 26 Oct 2021.

UN General Assembly (2019). United Nations decleration on the rights of peasants and other people working in rural areas. Resolution – adopted by the genral assembly. https://digitallibrary.un.org/record/1661560 . Zugegriffen: 02. Dez. 2021.
